# Supplementary material for: Evaluation of Nutritional and Health Status in Captive Eastern Indigo Snakes (Drymarchon couperi) in Response to Formulated Sausage Diet
Source: Animals (Basel). 2024 Nov 19;14(22):3324. doi: 10.3390/ani14223324 (PMC11591334; doi:10.3390/ani14223324)

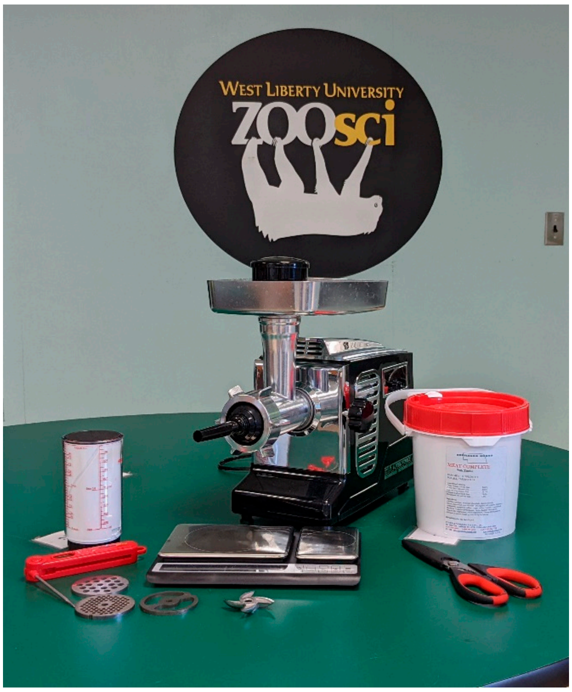

Equipment and tools used in making sausage diets (excluding meat, casings, and dicalcium phosphate/sodium chloride supplements).

How To: Whole Prey and Experimental Sausage Diet Preparation

Sausage diets are an opportunity to dial-in on the nutritional needs of carnivorous reptiles with specialized diets through whole prey, food-grade muscle meat, and/or supplements. The recipes presented in this document include a whole-prey sausage with equal proportions of commonly available whole prey items (adult and juvenile rodents, chicks, frog legs) and a 'faux-snake' sausage of pork, rabbit, alligator muscle meats composed of a similar nutrient profile to snakes predated by eastern indigo snakes (*Drymarchon couperi*) as reported by Dierenfeld et al. (2015; https://doi.org/10.1656/058.014.0311).

Before making sausages, the meat and machinery (meat hopper, screw, grinding head, cutting blades, grinding and stuffing plates) must be frozen overnight to avoid overheating the equipment. It is recommended to make batches of 1.3 kg, but batches of up to 2.2kg are possible if the meat and machinery are kept cool. Once the temperature of the meat rises above 5˚C, the meat should be placed back in the fridge to chill. Once meat has been ground, it should be stuffed that same day to avoid issues with texture or food safety/temperature ranges.

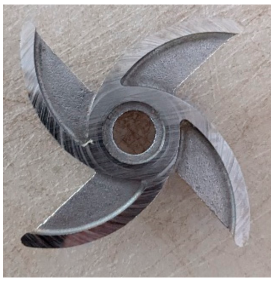

Cutting blades should be sharp to reduce friction and strain on the motor. If not properly oriented, the grinding plates and stuffing tubes will not sit flush in the body of the grinder.

1. Assemble the meat grinder according to the manual and check that the cutting blades are properly oriented. The STX International TurboForce 3000 electric meat grinder has an optional foot pedal to turn it on/off, but there are also buttons on the side that control the motor strength.
2. With a butcher knife, cut the meat into 2.5 cm cubes. This reduces the strain on the motor and prevents the meat from overheating. Excess fat should be stripped from any cut of meat.

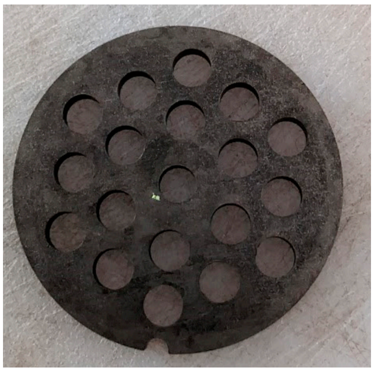

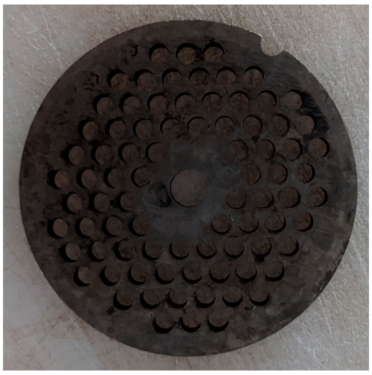

8 mm (left) and **4 mm (right)** plates used to ground meat. Gradually decreasing the size of the plate helps reduce friction and strain on the motor.

3. Using the high-power setting, push meat through the hopper with a tool and press the pedal (or button) to coarsely grind the meat with an **8 mm plate**.
4. Using the low-power setting, push meat through the hopper with a tool and press the pedal (or button) to finely grind the meat with a **4 mm plate**.

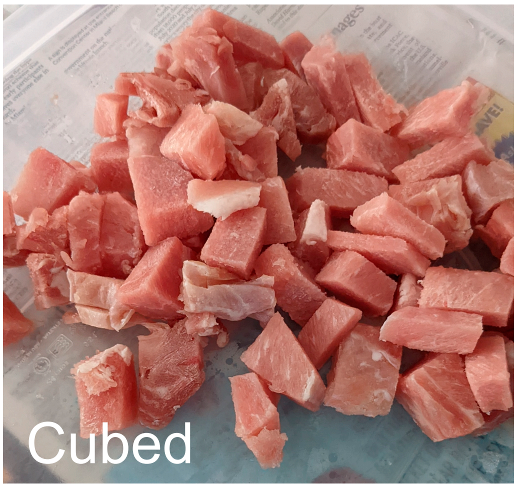

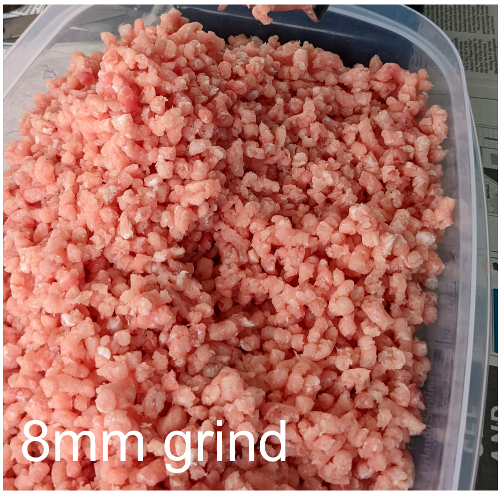

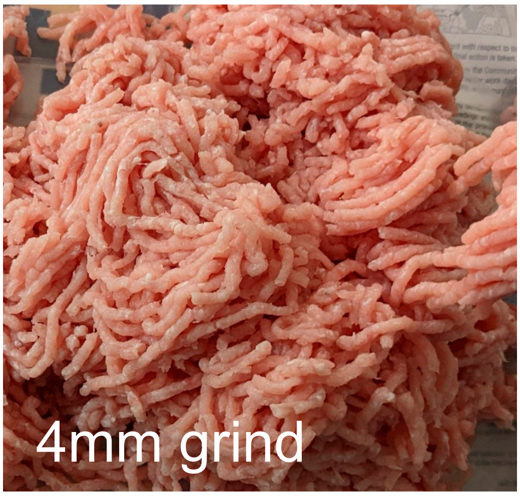

5. Weigh ground meat, then add water and supplements.
    a. **Whole Prey Diet:** 0.5 oz of water is added for every 10oz of meat.
    b. **Faux-Snake Diet:** 1oz of water is added for every 10oz of meat.
        i. Add Supplements for Experimental Sausage.
            1. For a .45 kg batch of snake sausage:
                a. 7.5g MeatComplete
                b. 10g Dicalcium phosphorus
                c. 1.75g Uniodized Salt
        ii. Mix THOROUGHLY with gloved hands.

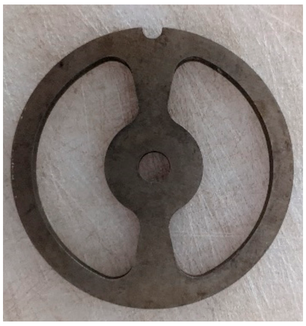

Wagon-wheel grinding plate for stuffing sausage.

6. Sausages are stuffed with ‘wagon wheel’ grinding plate – the cutting blade should also be removed. The diameter of casing should fit snugly around the stuffing tube.
7. Using the low-power setting, press the pedal (or button) and use a tool to push meat into the hopper. As the meat is pushed into the casing, hold it firmly and slowly pull it away from the meat grinder.

8. At the desired size, cut an additional 3 inches of casing away to tie-off the sausage.
    a. Pinch the casing above the filling, twist 2-3 times, and tie 1-2 knots. The sausage casing should feel firm but not tight. If it is too tight it could rupture while being frozen or fed out. If it is too loose the sausage will not hold its shape.
9. Cut off the excess casing, leaving space between the knot and where you’re cutting so that the knot does not become undone during the freezing process as the meat expands.
10. At the end of the batch when there isn’t enough meat in the hopper, stop using the grinder. Turn off the machine, unplug it from the wall, and disassemble it.
    a. Using a gloved hand or the end of a utensil wrapped in plastic, push the remaining meat through the stuffing tube and into the casing.
    b. Alternatively: for unfussy snakes or other carnivores, meat can be shaped into single-serving patties or molds and frozen.

Recipes

I recommend working with larger batches to reduce waste from leftover meat; a 2.2kg batch will take approximately 5 hours to process from start to finish.

Whole Prey Sausage. Determine the amount of sausage needed based on the feeding schedule/amount and number of snakes. Try to use equal amounts of each prey item (except QC/SM).

1. Large Mice
2. Small Rat
3. Chick
4. Quail chick + Small Mice
5. Frog Legs.

For a 1.8kg batch, the weights are…

1. 370g AM
2. 370g SR
3. 370g C
4. 185g QC
5. 185g SM
6. 370g FL

Experimental Sausage. Whole/processed rabbit carcasses must be processed prior to use; you can process approximately 1.5lbs of meat (no fat, connective tissue, or organs) from a 2lbs rabbit. When available, rabbit tenderloins are ideal for saving time and labor. For a 425g batch, the recipe is as follows.

1. 300g Pork Loin
2. 100g Rabbit
3. 25g Alligator filet
4. <u>Supplements:</u>
    a. 7.5g MeatComplete
    b. 10g Dicalcium phosphorus
    c. 1.75g Uniodized Salt

<u>Thawing & Feeding</u>

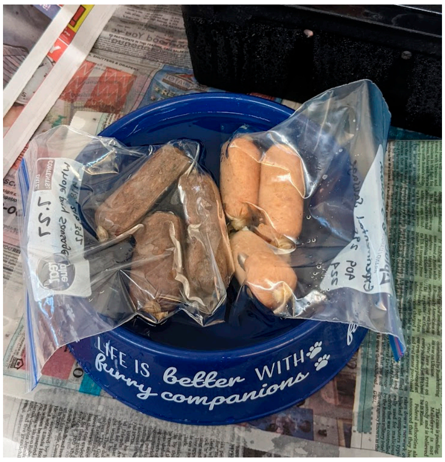

Thaw the sausage by placing the bag in a tub of water. If you notice water pooling in the bag, you can pour it out so that the sausages don't soak up too much water. This will degrade the integrity of the casing and increase the likelihood of a rupture.

When feeding the sausage out, hold it with the tongs so that it is parallel with the ground. If you let it dangle, it may rip or burst when struck by the snake. If you notice that the sausage has split casing, an opened end, or broke in half (or a batch is frequently bursting), place the sausage in a bowl or on top of newspaper and see if the snake will still eat it.

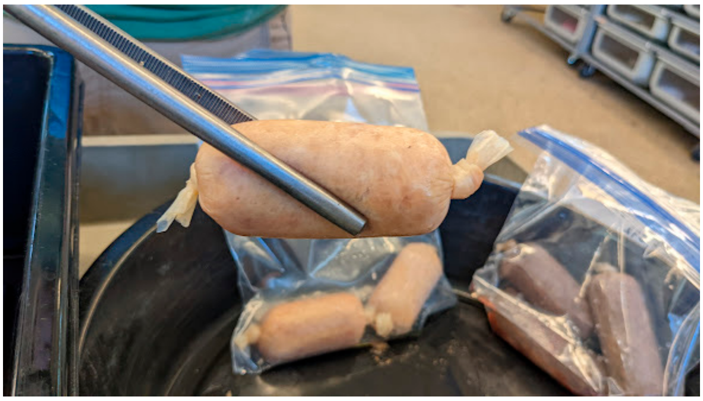

Supplement: Supplementary file 1 [file animals-14-03324-s001.zip › Jackson 2024 Sausage Protocols and Recipes.pdf]
