# Supplementary figures and images for: Evaluation of Nutritional and Health Status in Captive Eastern Indigo Snakes (Drymarchon couperi) in Response to Formulated Sausage Diet
Source: Animals (Basel). 2024 Nov 19;14(22):3324. doi: 10.3390/ani14223324 (PMC11591334; doi:10.3390/ani14223324)

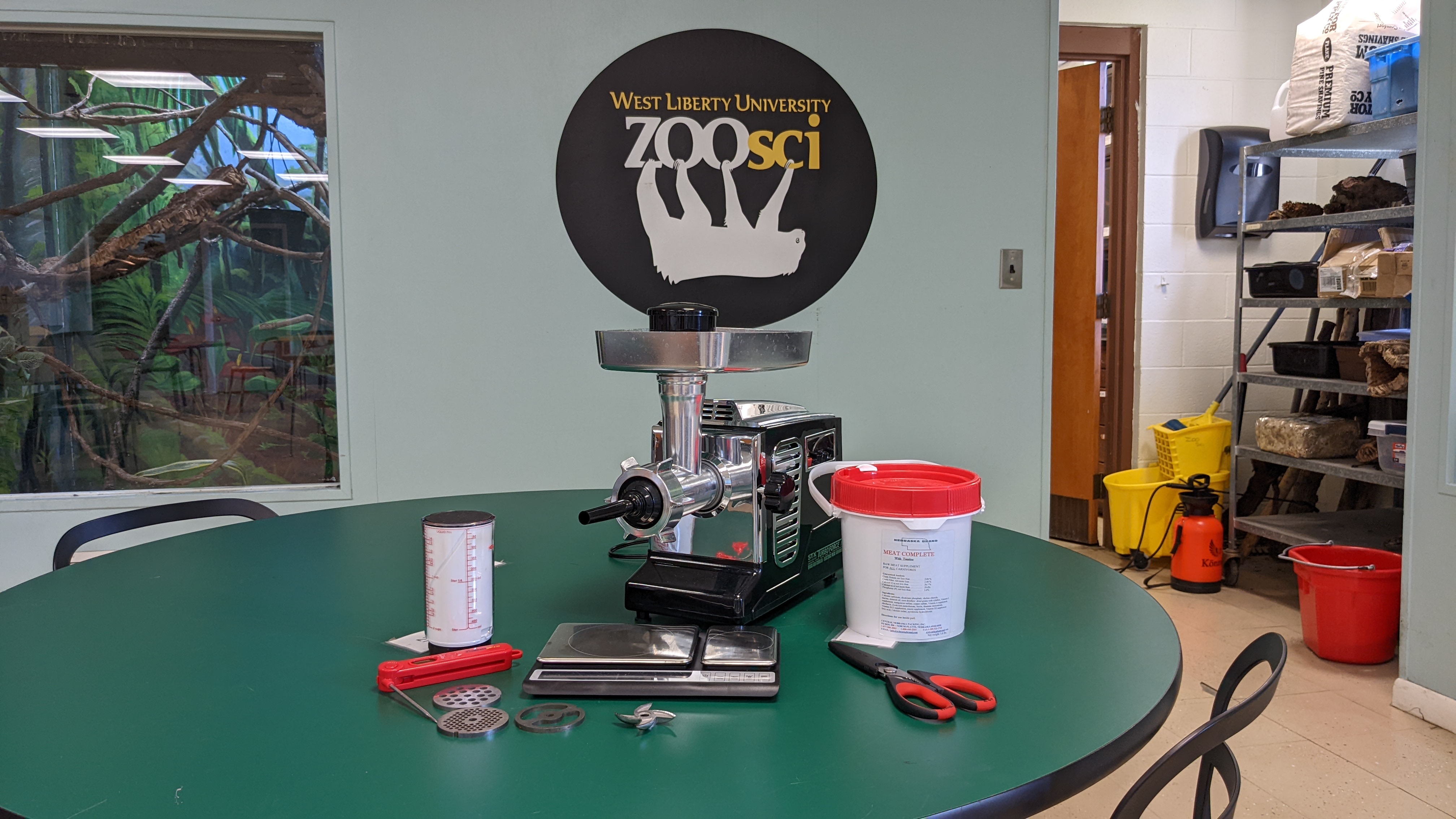

Supplement: Supplementary file 1 [file animals-14-03324-s001.zip › Protocol Images/SPR 1.jpg]

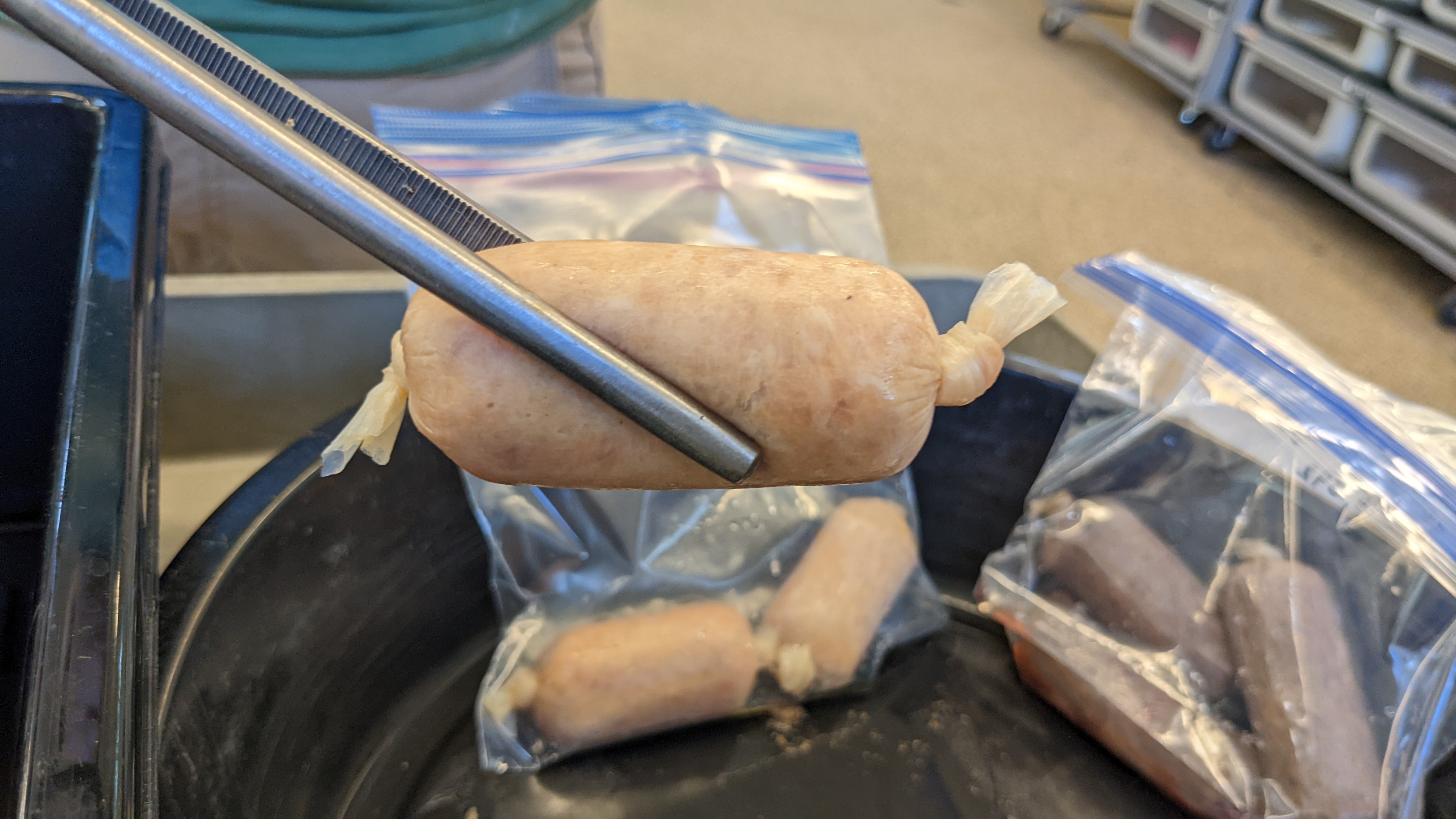

Supplement: Supplementary file 1 [file animals-14-03324-s001.zip › Protocol Images/SPR 10.jpg]

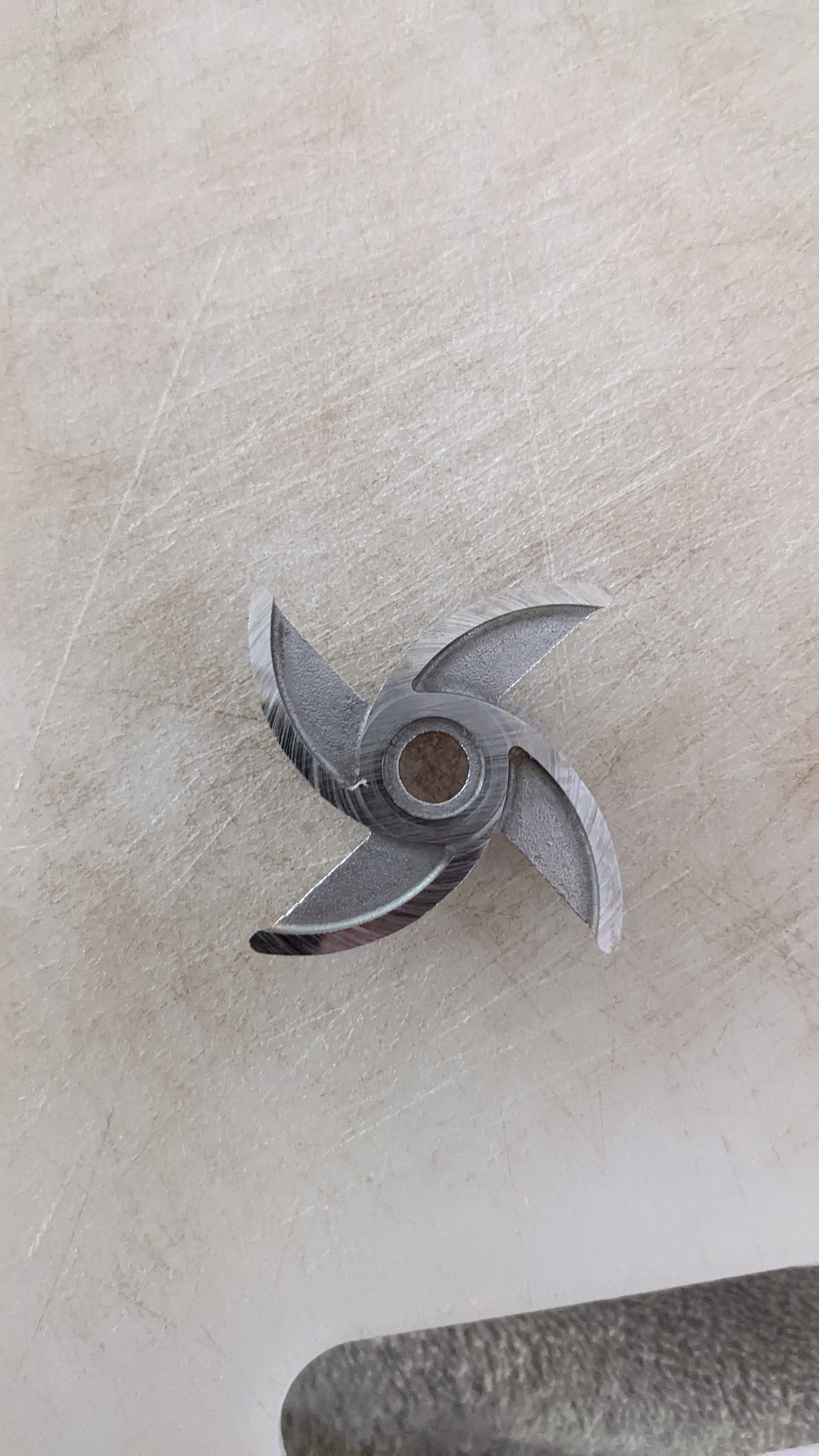

Supplement: Supplementary file 1 [file animals-14-03324-s001.zip › Protocol Images/SPR 2.jpg]

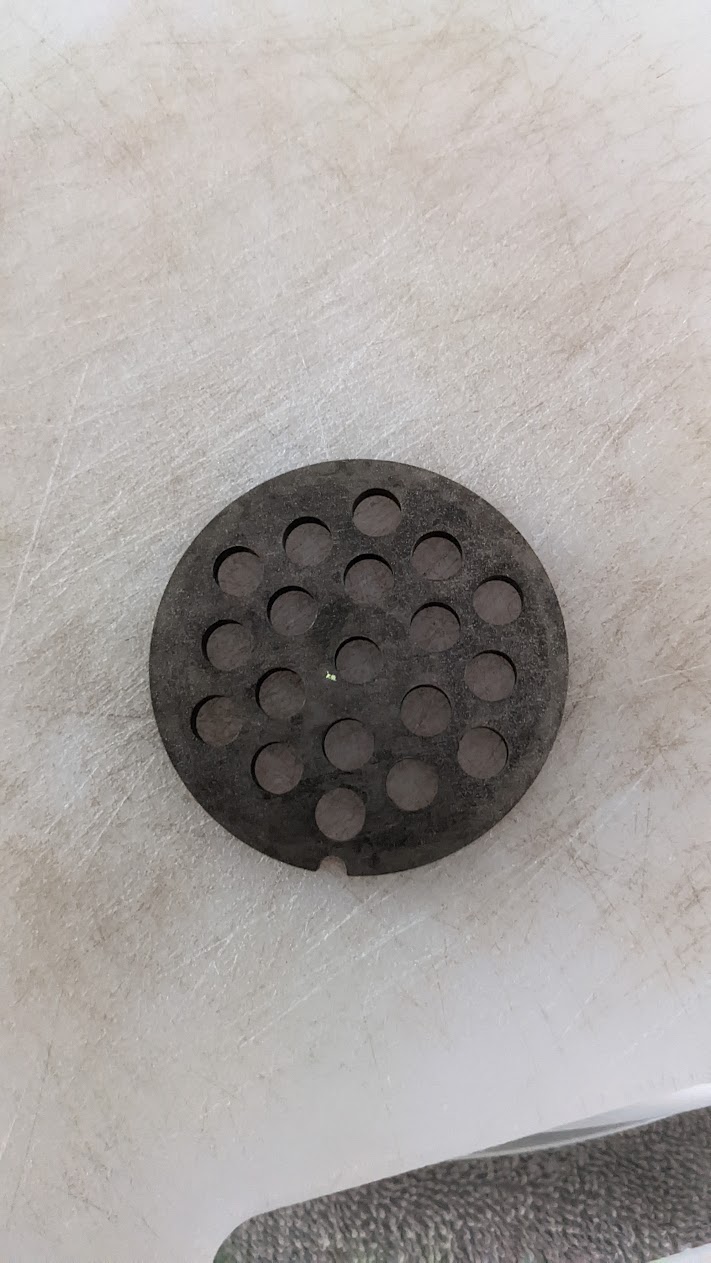

Supplement: Supplementary file 1 [file animals-14-03324-s001.zip › Protocol Images/SPR 3.jpg]

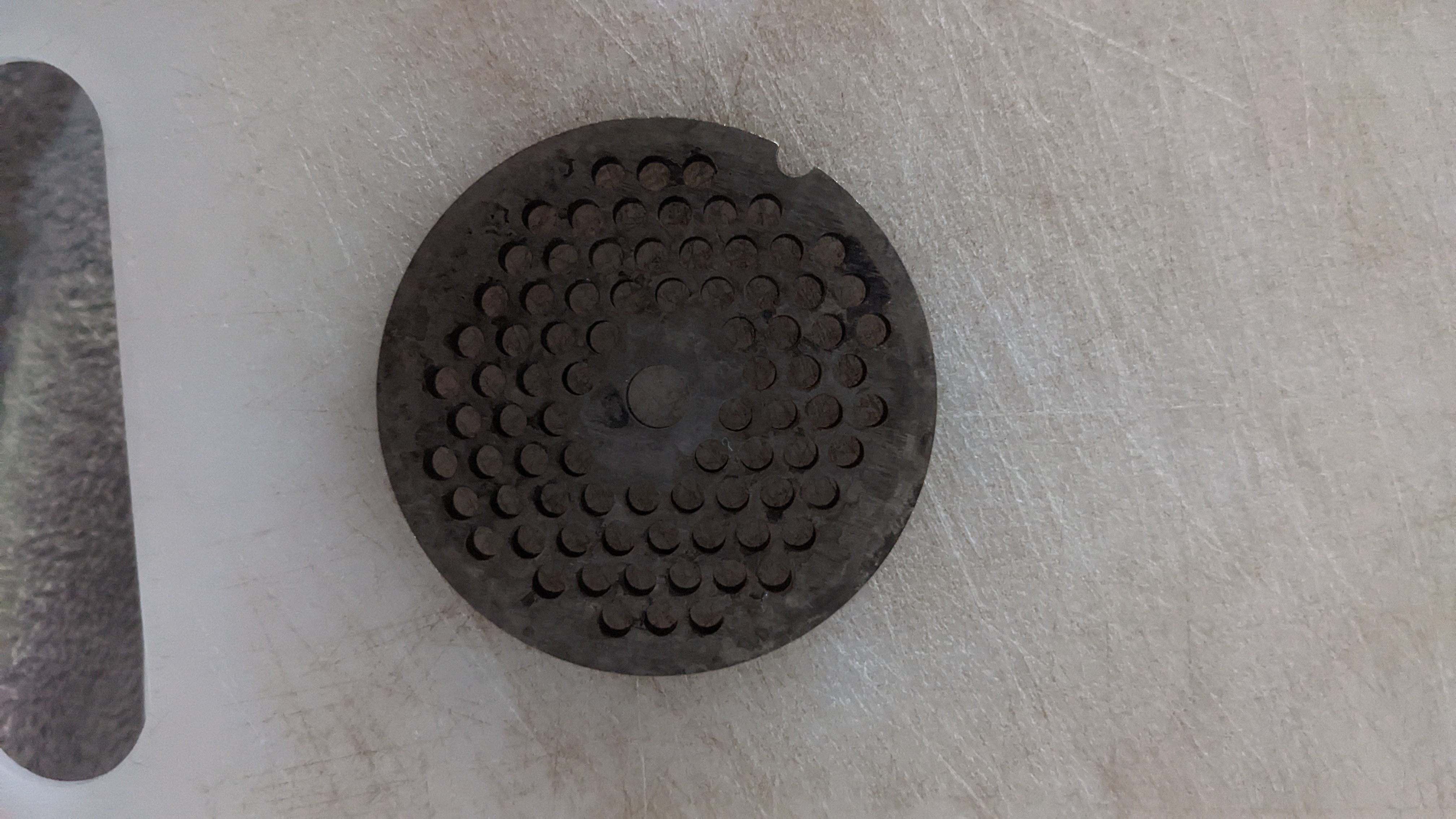

Supplement: Supplementary file 1 [file animals-14-03324-s001.zip › Protocol Images/SPR 4.jpg]

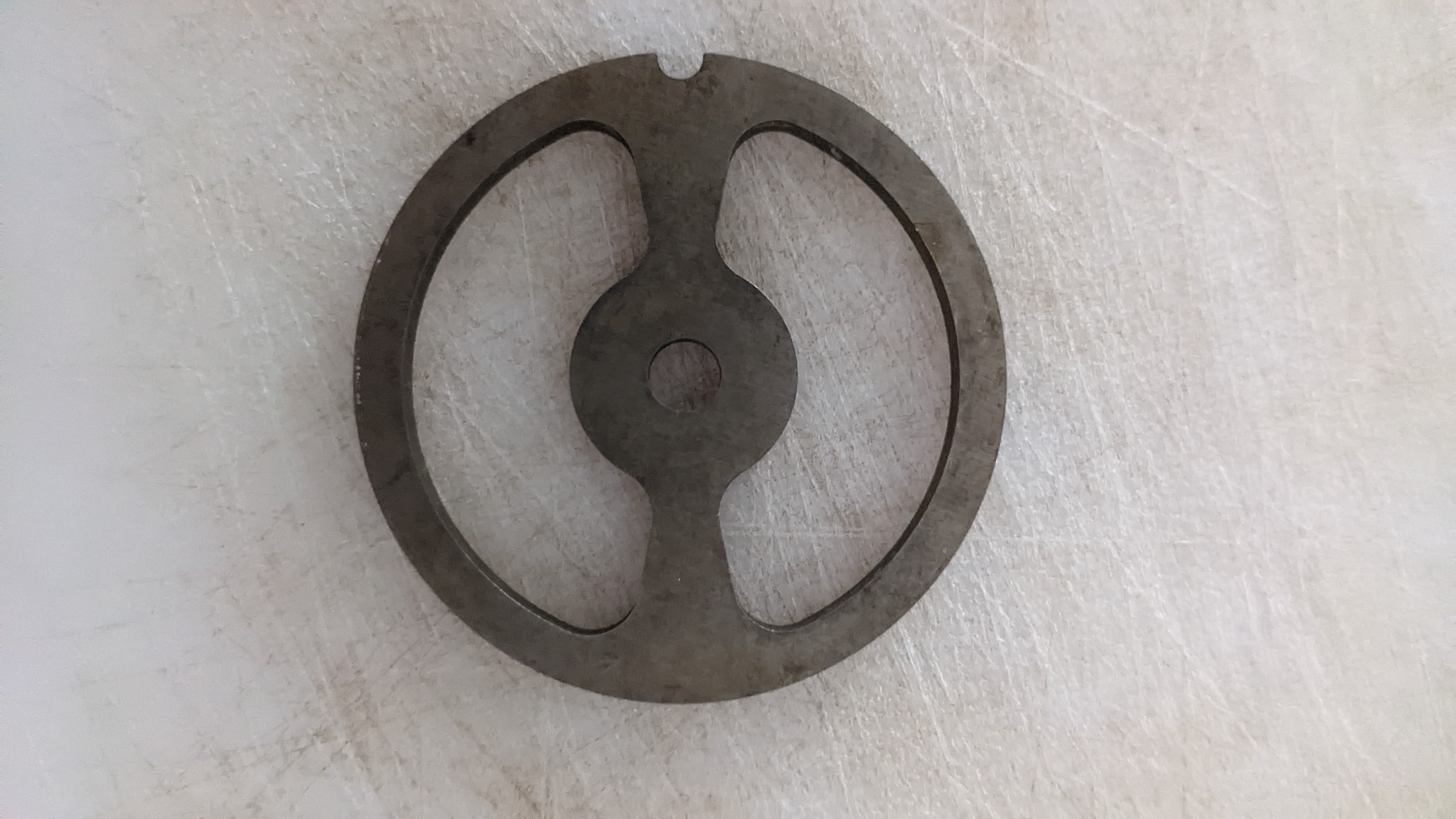

Supplement: Supplementary file 1 [file animals-14-03324-s001.zip › Protocol Images/SPR 5.jpg]

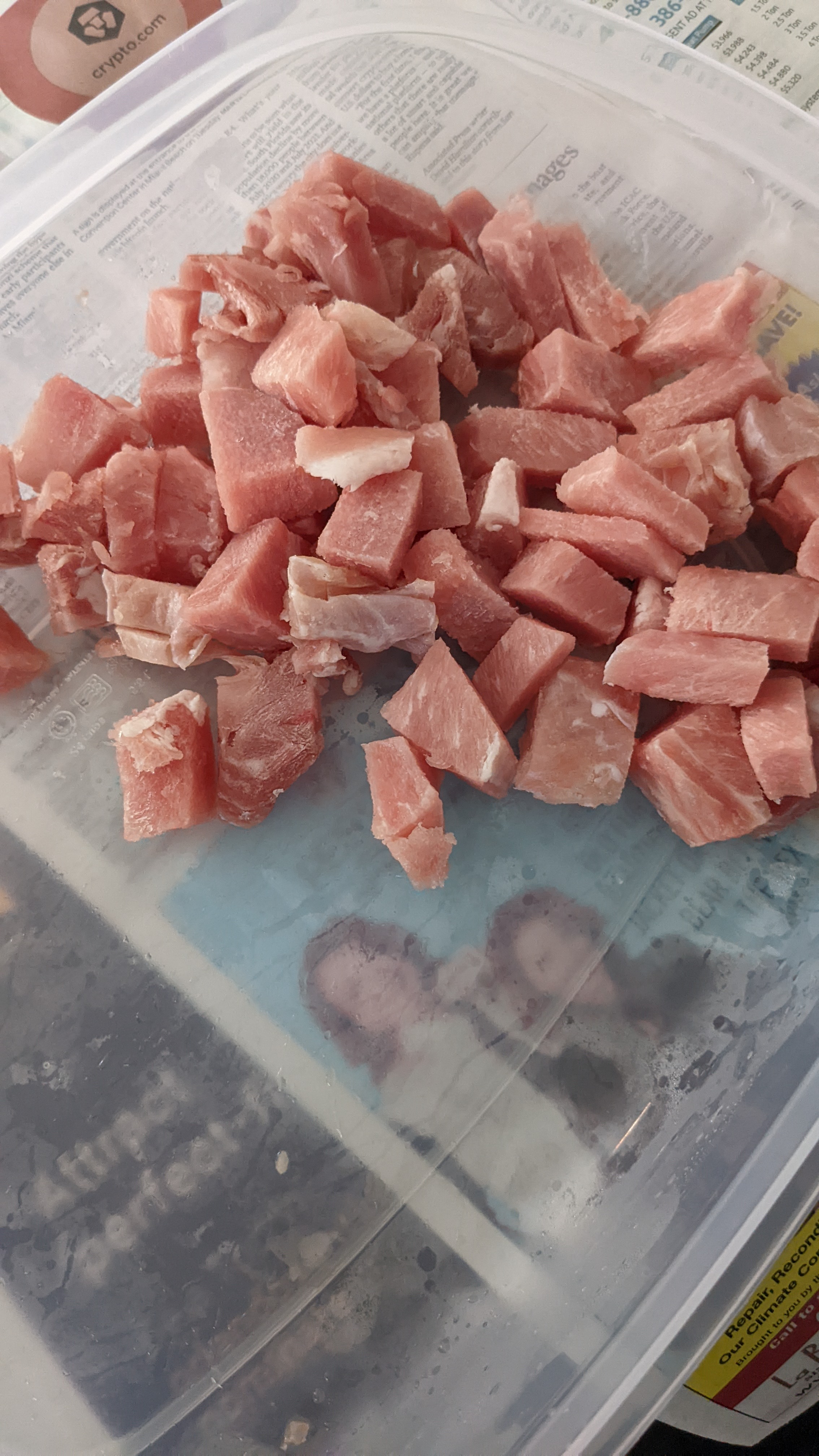

Supplement: Supplementary file 1 [file animals-14-03324-s001.zip › Protocol Images/SPR 6.jpg]

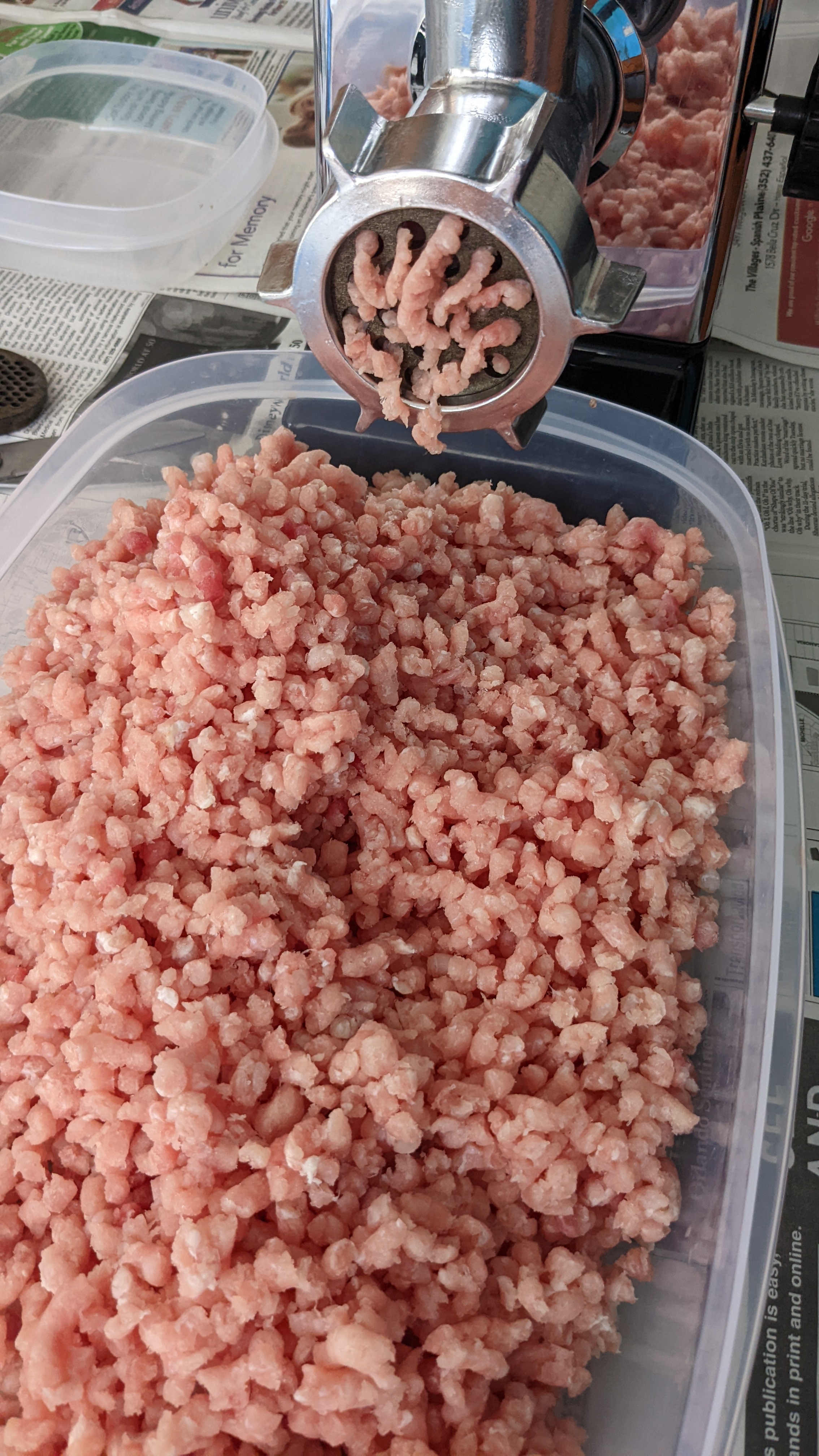

Supplement: Supplementary file 1 [file animals-14-03324-s001.zip › Protocol Images/SPR 7.jpg]

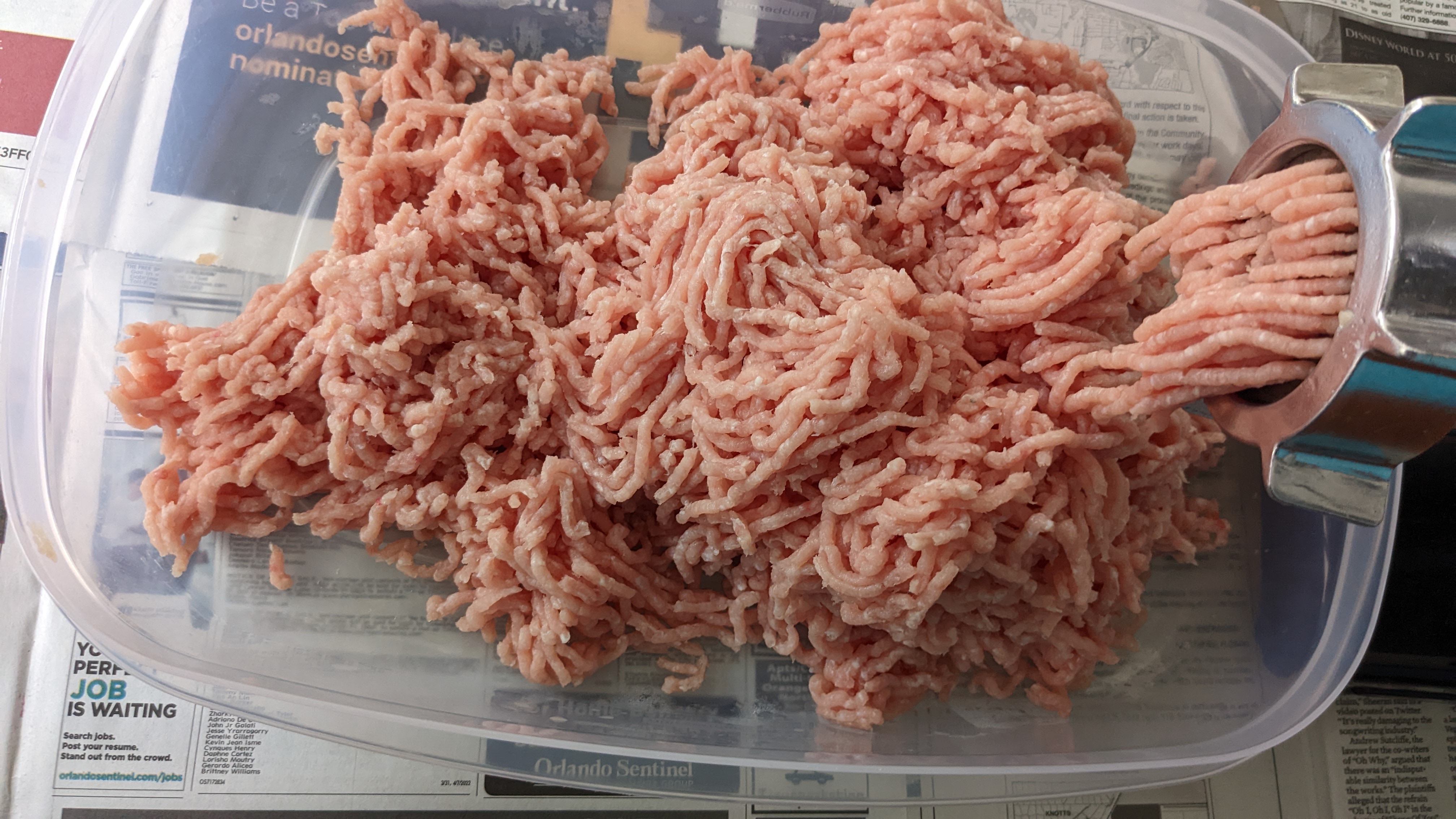

Supplement: Supplementary file 1 [file animals-14-03324-s001.zip › Protocol Images/SPR 8.jpg]

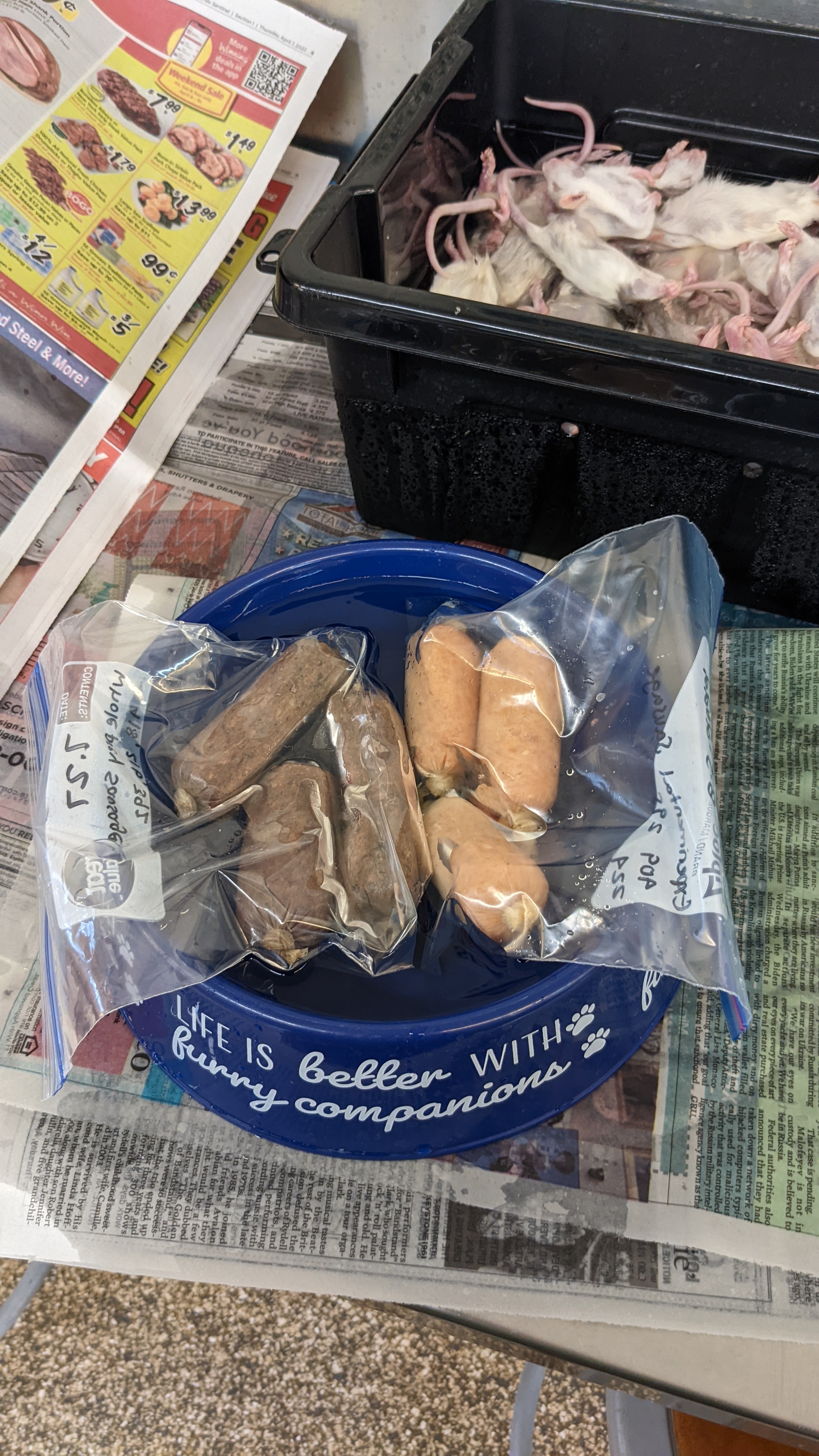

Supplement: Supplementary file 1 [file animals-14-03324-s001.zip › Protocol Images/SPR 9.jpg]
